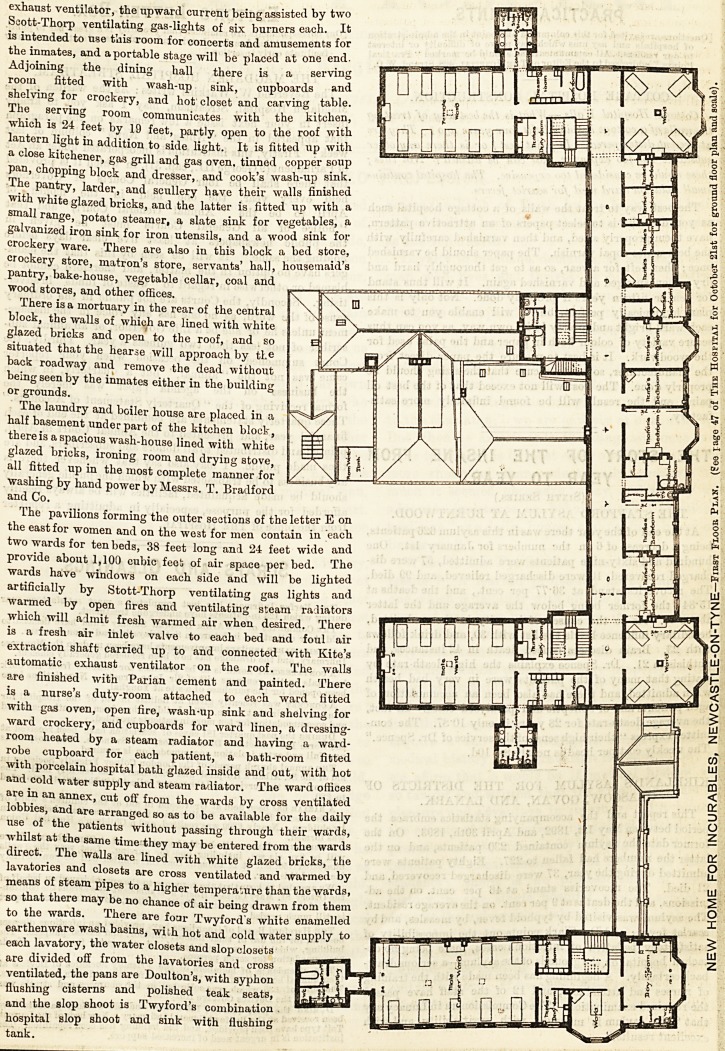# New Home for Incurables, Newcastle-On-Tyne

**Published:** 1893-11-11

**Authors:** 


					HOSPITAL CONSTRUCTION.
NEW HOME FOR INCURABLES, NEWCASTLE-
ON-TYNE,
The principal entrance is in the centre of the facade under
a moulded stone archway, surmounted by the city arms
forcing an open porch which admits to an entrance hall
paved with Minton's tiles of ornamental design. There is a
steam radiator in the hall to warm the air and prevent a cold
draught into the main corridor, which is approached through
a well designed screen, the sashes of which are filled in with
ornamental lead fret glazing by Messrs. Atkinson Brothers,
of Newcastle. On the right is the matron's sitting room
with linen store and officers' dining room adjoining, and be-
yond the women's workroom and dayroom with the
entrance for women at the east end of main corridor protected
by a glazed screen.
On the left of the entrance hall are the committee room and
library, the doctor's room and dispensary, and lavatory
adjoining, and beyond the men's workroom and day-room,
their entrance on the south front, and smoke-room. These
rooms all face the south and overlook the orna-
mental grounds, they are all well lighted,
cheerful of aspect, and decorated iu light and
pleasant t nes of colour, and are connected together by the
main corridor, which is 9 feet wide and 195 feet long.
There is a similar corridor on the first floor and both are
fireproof, the floors being formed with cement concrete filled
in between light steel joists and paved with a diagonal pattern
in red tiles and cement. The first floor of the main building
over the rooms before enumerated contains one double bedded
room and eight single bed-rooms for nurses, their sitting-
room when off duty, matron's bed-room, bath-room, and
lavatory en suite, and two dormitories or wards for inmates
of four beds each, and in the rear the officers' staircase,
nurses' bath-room, slop shoot, and other offices. The centre
block of the main building is raised to form attics for servants.
There is a hospital lift for the use of infirm patients con-
necting the different floors.
In the centre and behind the main building is the dining
hall and kitchen block. The dining hall opens out of the main
corridor near the front entrance, and is 36 feet long, 24 feet
wide, and 13 feet high, the walls are finished with parian
cement painted a light tint of green, and the wood work a rich
warm red. The ceiling is panelled with moulded ribs and
" finished to a wall with a richly moulded modilion cornice.
The dining hall is warmed by an open fire and two ventilating
steam radiators ; there are six inlets for the admsssion of
cold fresh air, and the foul air will be extracted through
shafts in the ceiling communicating with Kite's automatic
exhaust ventilator, the upward current being assisted by two
Scott-Thorp ventilating gas-lights of six burners each. It
is intended to use this room for concerts and amusements for
the inmates, and a portable stage will be placed at one end.
Adjoining the dining hall there is a serving
room fitted with wash-up sink, cupboards and
shelving for crockery, and hot closet and carving table.
The serving room communicates with the kitchen,
v\hich is 2-1 feet by 19 feet, partly open to the roof with
antern light in addition to side light. It is fitted up with
a close kitchener, gas grill and gas oven, tinned copper soup
pan, chopping block and dresser, and cook's wash-up sink.
he pantry, larder, and scullery have their walls finished
with white glazed bricks, and the latter is fitted up with a
small range, potato steamer, a slate sink for vegetables, a
galvanized iron sink for iron utensils, and a wood sink for
crockery ware. There are also iu this block a bed store,
crockery store, matron's store, servants' hall, hoxisemaid's
-nnt? U_1
pantry, bake-house, vegetable cellar, coal and
wood stores, and other offices.
There is a mortuary in the rear of the central
block, the walls of which are lined with white
glazed bricks and open to the roof, and so
situated that the hearse will approach by the
back roadway and remove the dead without
being seen by the inmates either in the building
or grounds.
The laundry and boiler house are placed in a
half basement under part of the kitchen block,
there is a spacious wash-house lined with white
glazed bricks, ironing room and drying stove,
all fitted up in the most complete manner for
washing by hand power by Messrs. T. Bradford
and Co.
The pavilions forming the outer sections of the letter E on
the east for women and on the west for men contain in each
two wards for ten beds, 38 feet long and 24 feet wide and
provide about 1,100 cubic feet of air space per bed. The
wards have windows on each side and will be lighted
artificially by Stott-Thorp ventilating gas lights and
warmed by open fires and ventilating steam radiators
which will admit fresh warmed air when desired. There
is a fresh air inlet valve to each bed and foul air
extraction shaft carried up to and connected with Kite's
automatic exhaust ventilator on the roof. The walls
are finished with Parian cement and painted. There
is a nurse's duty-room attached to each ward fitted
with gas oven, open fire, wash-up sink and shelving for
ward crockery, and cupboards for ward linen, a dressing-
room heated by a steam radiator and having a ward-
robe cupboard for each patient, a bath-room fitted
with porcelain hospital bath glazed inside and out, with hot
and cold water supply and steam radiator. The ward offices
are in an annex, cut off from the wards by cross ventilated
lobbies, and are arranged so as to be available for the daily
use of the patients without passing through their wards,
whilst at the same time they may be entered from the wards
direct. The walls are lined with white glazed bricks, the
lavatories and closets are cross ventilated and warmed by
means of steam pipes to a higher temperature than the wards,
so that there may be no chance of air being drawn from them
to the wards. There are four Twyford s white enamelled
earthenware wash basins, wilh hot and cold water supply to
pn.nVl lmrn+rvrtr tVio ' " "
eacli lavatory, the water closets and slop closets
are divided off from the lavatories and cross
ventilated, the pans are Doulton's, with syphon
flushing cisterns and polished teak seats,
and the slop shoot is Twyford's combination
hospital slop shoot and sink with flushing
tank.
exhaust ventilator, the upward current being assisted by two
Scott-Thorp ventilating gas-lights of six burners each. It
is intended to use this room for concerts and amusements for
the inmates, and a portable stage will be placed at one end.
Adjoining the dining hall there is a serving
room fitted with wash-up sink, cupboards and
shelving for crockery, and hot closet and carving table.
The serving room communicates with the kitchen,
which is 24 feet by 19 feet, partly open to the roof with
lantern light in addition to side light. It is fitted up with
a close kitchener, gas grill and gas oven, tinned copper soup
pan, chopping block and dresser, and cook's wash-up sink.
The pantry, larder, and scullery have their walls finished
with white glazed bricks, and the latter is fitted up with a
small range, potato steamer, a slate sink for vegetables, a
galvanized iron sink for iron utensils, and a wood sink for
crockery ware. There are also in this block a bed store,
crockery store, matron's store, servants' hall, housemaid's
pantry, bake-house, vegetable cellar, coal and
wood stores, and other offices.
There is a mortuary in the rear of the central
block, the walls of which are lined with white
glazed bricks and open to the roof, and so
situated that the hearse will approach by the
back roadway and remove the dead without
being seen by the inmates either in the building
or grounds.
The laundry and boiler house are placed in a
half basement under part of the kitchen block,
there is a spacious wash-house lined with white
glazed bricks, ironiDg room and drying stove,
all fitted up in the most complete manner for
washing by hand power by Messrs. T. Bradford
and Co.
The pavilions forming the outer sections of the letter E on
the east for women and on the west for men contain in each
two wards for ten beds, 38 feet long and 24 feet wide and
provide about 1,100 cubic feet; of air space per bed. The
wards have windows on each side and will be lighted
artificially by Stott-Thorp ventilating gas lights and
warmed by open fires and ventilating steam radiators
which will admit fresh warmed air when desired. There
is a fresh air inlet valve to each bed and foul
extraction shaft carried up to and connected with Kite's
automatic exhaust ventilator on the roof. The walls
are finished with Parian cement and painted. There
is a nurse's duty-room attached to each ward fitted
with gas oven, open fire, wash-up sink and shelving for
ward crockery, and cupboards for ward linen, a dressing-
room heated by a steam radiator and having a ward-
robe cupboard for each patient, a bath-room fitted
with porcelain hospital bath glazed inside and out, with hot
and cold water supply and steam radiator. The ward offices
are in an annex, cut off from the wards by cross ventilated
lobbies, and are arranged so as to be available for the daily
xise of the patients without passing through their wards,
whilst at the same time they may be entered from the wards
direct. The walls are lined with white glazed bricks, the
lavatories and closets are cross ventilated and warmed by
means of steam pipes to a higher temperature than the wards,
so that there may be no chance of air being drawn from them
to the wards. There are four Twyford s white enamelled
earthenware wash basins, wuh hot and cold water supply to
each lavatory, the water closets and slop closets
are divided off from the lavatories and cross
ventilated, the pans are Doulton's, with syphon
flushing cisterns and polished teak seats,
and the slop shoot is Twyford's combination
hospital slop shoot and sink with flushing
tank.

				

## Figures and Tables

**Figure f1:**